# Visual Non-Instrumental On-Site Detection of Fumonisin B_1_, B_2_, and B_3_ in Cereal Samples Using a Clean-Up Combined with Gel-Based Immunoaffinity Test Column Assay

**DOI:** 10.3390/toxins10040165

**Published:** 2018-04-19

**Authors:** Wei Sheng, Hesen Wu, Weihong Ji, Zhi Li, Fangyu Chu, Shuo Wang

**Affiliations:** 1 State Key Laboratory of Food Nutrition and Safety, Key Laboratory of Food Nutrition and Safety, Ministry of Education of China, College of Food Engineering and Biotechnology, Tianjin University of Science and Technology, Tianjin 300457, China; wuhesen@gz.gov.cn (H.W.); jiweihongsky@163.com (W.J.); lizhi3204@126.com (Z.L.); cfy0809@163.com (F.C.); 2 Beijing Advanced Innovation Center for Food Nutrition and Human Health, Beijing Technology and Business University (BTBU), Beijing 100048, China

**Keywords:** fumonisins, immunoaffinity test column, clean-up column, visual non-instrumental detection, cereal samples

## Abstract

A visual immunoaffinity test column (IATC) assay was developed to detect fumonisins in cereal samples for spot tests without the need for special instruments. The developed IATC assay had equivalent recognition capability for fumonisin B_1_ (FB_1_), fumonisin B_2_ (FB_2_), or fumonisin B_3_ (FB_3_), and exhibited no cross-reactivity with aflatoxin B_1_, ochratoxin A, zearalenone, or the T-2 toxin. The sample pretreatment was accomplished more rapidly and with greater ease, the entire assay procedure was completed in approximately 10 min, including sample pretreatment and testing. The limits of detection (LODs) of the IATC assay to detect fumonisins in the maize, barley, oat, and millet samples were 20 μg kg^−1^. The results of the spiked maize, barley, oat, and millet and real maize samples by the IATC assay agreed well with the results obtained by the commercial fumonisin enzyme-linked immunosorbent assay (ELISA) test kit and liquid chromatography-tandem mass spectrometry (LC-MS/MS), respectively. The developed IATC assay can serve as a useful screening tool for the rapid, qualitative, and semi-quantitative detection of the total content of fumonisins (sum of FB_1_, FB_2_, and FB_3_) in cereal samples on-site.

## 1. Introduction

*Fusarium verticillioides* and *Fusarium proliferatum* are the main *Fusarium* species producing fumonisins, which contaminate mainly maize and maize products. Fumonisins were first isolated from maize by Gelderblom in 1988 [1]. Fumonisin B (FB) is the main contaminant of natural cereal samples, with fumonisin B_1_ accounting for approximately 70% of fumonisins; and fumonisin B_2_ and fumonisin B_3_ for approximately 20% and 10% [2], respectively. To date, FBs have been found in foodstuffs, such as cereals [3], dried figs [4], pine nuts [5], coffee [6], black tea, and medicinal plants [7]. Many studies have shown that FBs cause a variety of diseases in animals and humans, such as brain lesions in horses [8], porcine pulmonary edema syndrome [9], and liver and kidney diseases in mammals [10]. Considering its toxicity, many countries and institutions have established limits for FBs in agricultural products. For example, the maximum levels of total FBs (FB_1_ + FB_2_ + FB_3_) established by the U.S. Food and Drug Administration [11]: 2–4 mg kg^−1^ and 5–100 mg kg^−1^ in human food and animal feed, respectively. The Scientific Committee on Food of European Commission determined that the human tolerable daily intake (TDI) for FBs (FB_1_ + FB_2_ + FB_3_) is 2 μg kg^−1^ body weight [12].

Conventional methods for detecting FBs mainly involve chromatography and immunoassay. The most used chromatographic method for the determination of fumonisins is high performance liquid chromatography coupled with fluorescence detection, after pre-column derivatization with *o*-phthaldialdehyde (OPA) reagent [13,14,15]. A few of chromatographic methods, such as liquid chromatography tandem mass spectrometry [16] and gas chromatography-mass spectrometry [17], have also been reported to detect fumonisins. However, these techniques require the use of expensive apparatus, take a long time, and involve many skilled manipulations; therefore, chromatography analysis is limited in terms of the on-site detection of FBs in cereal samples. Compared with chromatography, immunoassay has the advantages of sensitivity, specificity, and simplicity. Indirect competitive enzyme-linked immunosorbent assay (ELISA) has been developed to detect FB_1_ [18]. Colloidal gold immunochromatographic test strips are used to determine fumonisin B_1_ in food and feedstuff samples, the LOD of the test strips is 50 μg L^−1^ [19]. Using a membrane-based flow-through immunoassay for the multi-detection of mycotoxins, the cut-off levels of 100, 1000, 1000, and 2500 μg L^−1^ (μg kg^−1^) have been achieved for a FB_1_ standard solution, wheat, maize, and silage, respectively [20].

The immunoaffinity test column (IATC) is a gel-based immunoassay derived from immunoaffinity chromatography [21]. This type of assay has been used to non-instrumentally analyze pyrene in water samples [22], ochratoxin A in beer [23], chloramphenicol in food samples [24], and clindamycin residues in milk [25]. The IATC can be combined with a clean-up column to analyze food samples with prominent matrix interference [26,27,28]. In our study, a rapid and non-instrumental IATC assay was developed to analyze fumonisins and a silica-C18 clean-up column was used to eliminate the matrix interference from cereals.

## 2. Results and Discussion

### 2.1. Development of IATC Assay

**Figure 1:**
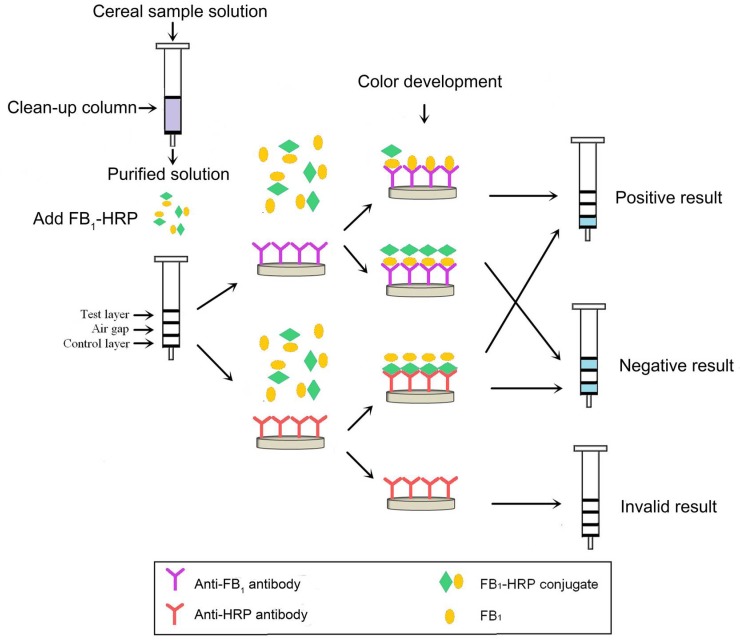
Test procedure schematic of the IATC assay. FB_1_: fumonisin B_1_. HRP: horseradish peroxidase.

**Figure 2:**
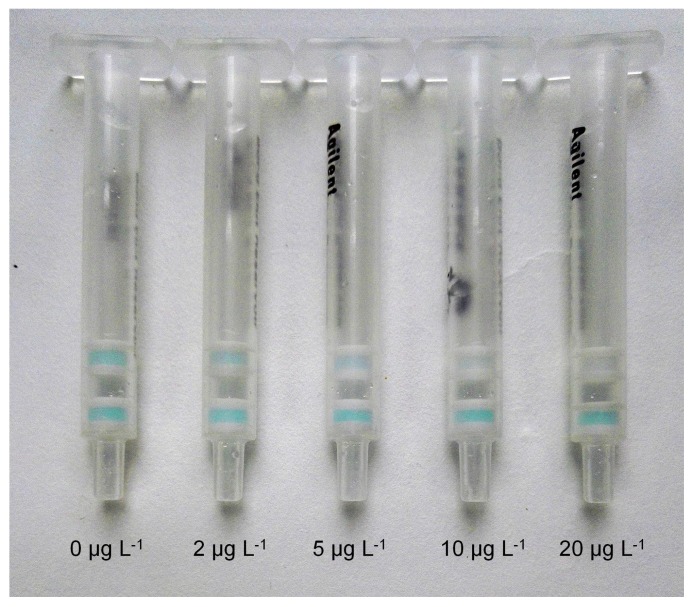
Detection of FB_1_ with the IATC using a series of dilutions (0, 2, 5, 10, and 20 μg L^−1^) of FB_1_ prepared in PBS (pH 7.4, 0.01 mol L^−1^).

The color of the test layer (the anti-FB_1_ antibody gel) conformed to the color of the control layer (the anti-horseradish peroxidase (anti-HRP) antibody gel) when the anti-FB_1_ antibody gel and the anti-HRP antibody gel were diluted with the blocked gel at ratios of 1:20 and 1:30, respectively. The optimal dilution ratio of the FB_1_-HRP was 4 × 10^5^-fold in phosphate-buffered saline (PBS, pH 7.4, 0.01 mol L^−1^). As shown in Figure 1, the FB_1_-HRP and FB_1_ in the sample solution could bond with the anti-FB_1_ antibody in the test layer gels competitively. As the concentration of FB_1_ in sample increased, the bond of FB_1_-HRP with anti-FB_1_ antibody decreased. This resulted in the color of the test layer becoming weaker, or even colorless. The visual limit of detection (LOD) for the IATC assay was considered the lowest concentration of FB_1_ at which the test layer showed no color. The blue color of the control layer indicated the reliability of the IATC assay. The assay was identified as invalid if the blue color did not develop in the control layer. The FB_1_ standards were diluted in PBS (pH 7.4, 0.01 mol L^−1^) to give various concentrations at 0, 2, 5, 10, and 20 μg L^−1^. As shown in Figure 2, along with the increase in concentration of FB_1_, the color of the test layer became weak. When the concentration of FB_1_ was 10 μg L^−1^, the test layer showed no color. The visual LOD for this developed IATC assay was 10 μg L^−1^ for FB_1_ in the assay buffer.

### 2.2. Specificity of IATC Assay

**Figure 3:**
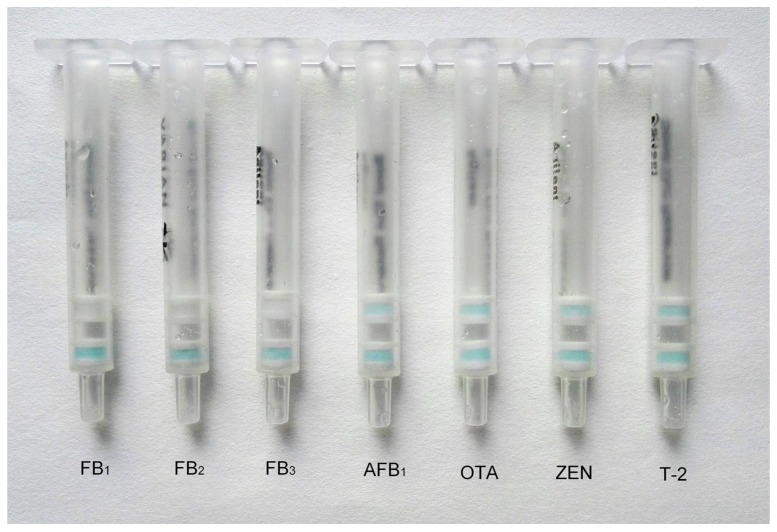
Specificity analysis of the IATC assay. 10 μg L^−1^ of FB_1_, 10 μg L^−1^ of FB_2_, 10 μg L^−1^ of FB_3_, 1000 μg L^−1^ of AFB_1_, 1000 μg L^−1^ of ochratoxin A (OTA), 1000 μg L^−1^ of zearalenone (ZEN), and 1000 μg L^−1^ of T-2 toxin standards were applied to the test.

In this study, FB_1_, FB_2_, FB_3_, AFB_1_, ochratoxin A (OTA), zearalenone (ZEN), and T-2 toxin standards were used to assess the specificity of the IATC assay. The concentrations of FB_1_, FB_2_, and FB_3_ were 10 μg L^−1^, and the concentrations of AFB_1_, OTA, ZEN, and T-2 toxin were 1000 μg L^−1^. The results of the IATC tests (Figure 3) showed that the color of the test layer and control layer was consistent for the high concentrations of AFB_1_, OTA, ZEN, and T-2 toxin, and the test layer was colorless at 10 μg L^−1^ for FB_1_, FB_2_, and FB_3_. Therefore, the developed IATC assay had good specificity and equivalent recognition capability for FB_1_, FB_2_, or FB_3_, and could be applied to detect the total concentration of FBs (FB_1_ + FB_2_ + FB_3_).

### 2.3. Sample Analysis

#### 2.3.1. Maize Samples

**Figure 4:**
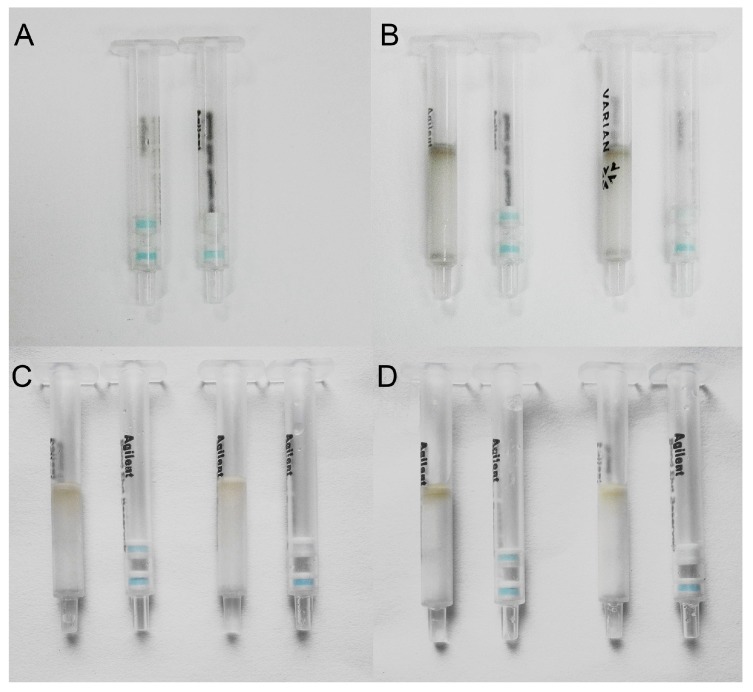
Analysis of the spiked cereal samples with the IATC. (**A**) Maize; (**B**) Barley; (**C**) Oat; and (**D**) Millet. The left column in each graph shows the result of the sample without FB_1_, and the right column in each graph shows the result of the sample spiked with 20 μg kg^−1^ of FB_1_.

A blank maize sample and spiked maize sample (20 μg kg^−1^) were extracted and 1 mL of the sample extracted solution mixed with FB_1_-HRP was analyzed using the IATC. The results showed (Figure 4A) that a blue color appeared in the test layer and the control layer at the same time when detecting the blank sample, while it was colorless in the test layer when detecting the spiked sample. This indicated that the method was feasible to extract the FB_1_ from the maize sample, and no matrix interference on the assay was observed. Finally, the visual LOD of FB_1_ in the maize sample using IATC was 20 μg kg^−1^.

#### 2.3.2. Barley Samples

**Figure 5:**
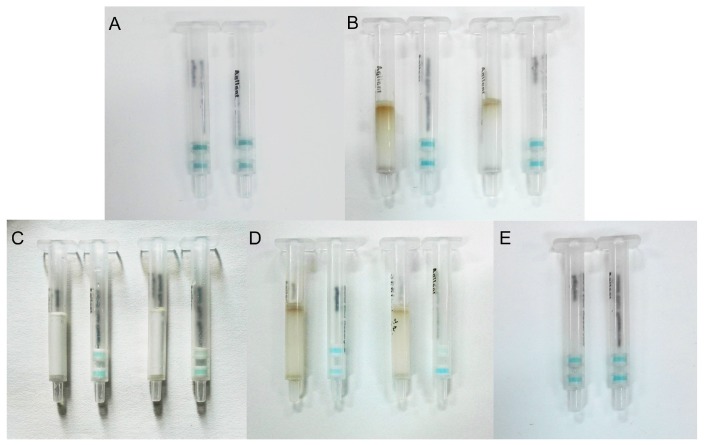
Eliminating the interference of samples matrix on assay. (**A**) Sample extracted solution without clean-up was analyzed by the IATC. (**B**) Sample extracted solution was flowed into the SAX clean-up column and analyzed by the IATC. (**C**) FB_1_ standard solution was flowed into the SAX clean-up column and analyzed by the IATC. (**D**) Samples with NaCl (2 g of samples with 0.5 g of NaCl) were extracted and the sample extracted solution was flowed into the SAX clean-up column and analyzed by the IATC. (**E**) Samples with NaCl (2 g of samples with 0.5 g of NaCl) were extracted and analyzed by the IATC. The left column in each graph shows the result of the sample without FB_1_, and the right column in each graph shows the result of the sample spiked with 20 μg kg^−1^ of FB_1_.

Blank barley and spiked barley samples (20 μg kg^−1^) were extracted and 1 mL of the sample extracted solution mixed with FB_1_-HRP was analyzed using the IATC. The result shown in Figure 5A depicts the blue color that appeared in both layers for the blank sample and the spiked sample, which indicated a false negative result for the spiked sample. The extract method has proven feasible to extract FB_1_ from a maize sample, so we considered that the barley sample extracted solution contained some impurities or proteins that may influence the IATC assay, and the barley sample extracted solution could not be directly tested by the IATC. Therefore, a clean-up column with different solid-phase materials was used to eliminate matrix interference.

The extracted solutions from the blank and spiked barley samples were flowed through the clean-up column (filled with strong anion exchange silica (SAX) sorbents), then 1 mL of purified sample solution was mixed with FB_1_-HRP and detected by the IATC. However, the test results still showed a false negative result when detecting the spiked sample: a blue color still appeared in the test layer (Figure 5B). We considered that the purified sample solution still contained some soluble proteins or the FB_1_ was absorbed by the clean-up column. Therefore, blank PBS solution and FB_1_ standard solution (10 μg L^−1^) were flowed through the SAX clean-up column and detected by the IATC where the test result showed the test layer was colorless for the FB_1_ standard solution (Figure 5C), which demonstrated that the SAX clean-up column did not absorb FB_1_. Therefore, we believe that the purified sample solution still contained some soluble proteins, which led to the test interference. A high concentration of sodium chloride was used to precipitate the soluble proteins in our study. Two grams of sample was added to 0.5 g NaCl and extracted using the extraction solution. The sample extracted solution was purified using the SAX clean-up column and the purified sample solution was mixed with FB_1_-HRP. One milliliter of the mixed solution was analyzed using the IATC. The result of the IATC assay (Figure 5D) showed that there was a significant color difference between the spiked sample and the blank sample in the test layer: the test layer appeared an approximately colorless result for the spiked sample. Therefore, we considered that adding a high concentration of sodium chloride and the use of the SAX clean-up column were indispensable to eliminate the matrix interference in the barley sample extracted solution. The blank barley sample and spiked sample were added to NaCl and extracted without clean-up. The assay result showed that a blue color appeared in the test layer for the spiked sample (Figure 5E), which indicated that the matrix interference was not eliminated by just adding a high concentration of sodium chloride.

**Figure 6:**
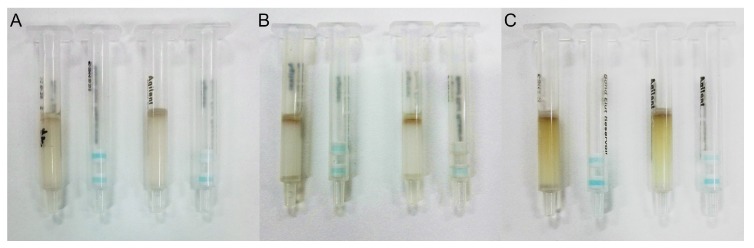
Selection of clean-up column. (**A**) SAX sorbent clean-up column; (**B**) silica-C18 sorbent clean-up column; and (**C**) NH_2_ sorbent clean-up column. The left column in each graph shows the result of the sample without FB_1_, and the right column in each graph shows the result of the sample spiked with 20 μg kg^−1^ of FB_1_.

We also chose other sorbents (silica-C18, NH_2_-derived silica) to fill the clean-up column and to purify the sample extracted solution. We found that the clean-up column filled with SAX sorbents, could eliminate most of the matrix interference (Figure 6A), but the trial using a clean-up column filled with silica-C18 showed that the silica-C18 eliminated the matrix interferences more completely (Figure 6B). A trial using a clean-up column filled with NH_2_-derived silica did not remove the matrix interferences (Figure 6C). Therefore, silica-C18 was chosen as the sorbent for the clean-up column for further experiments. As shown as Figure 4B, the visual LOD of the IATC assay to detect FB_1_ in the barley sample was 20 μg kg^−1^.

#### 2.3.3. Oat and Millet Samples

Significant matrix interference on the IATC assay was observed for the analysis of the oat and millet samples. The addition of a high concentration of sodium chloride and use of clean-up column were applied. A blank sample and spiked sample (20 μg kg^−1^) of oat and millet were extracted, then the extracted solution was passed through a clean-up column (filled with silica-C18), and 1 mL of the purified sample solution mixing with FB_1_-HRP was analyzed using the IATC. The IATC results of the oat and millet samples are shown in Figure 4C,D. The left test column in each graph shows the IATC assay result for the blank sample, and the right test column in each graph shows the IATC assay result for the spiked sample. Finally, the visual LODs of the IATC assay to detect FB_1_ in the oat and millet samples were 20 μg kg^−1^.

Due to the developed IATC assay had equivalent recognition capability for FB_1_, FB_2_, or FB_3_, and it can detect the total concentration of FBs (FB_1_ + FB_2_ + FB_3_). That is to say, the IATC assay will provide a positive result when the total concentration of FB_1_, FB_2_, and FB_3_ is equal to, or higher than 20 μg kg^−1^ (the visual LOD of FB_1_ in cereal samples). Therefore, the visual LOD of the IATC assay to detect total fumonisins in maize, barley, oat, or millet samples was 20 μg kg^−1^.

### 2.4. Comparison of IATC and Commercial ELISA Test Kits for the Analysis of Cereal Samples

The maize, barley, oat, and millet samples were certified as negative FB samples by liquid chromatography-tandem mass spectrometry (LC-MS/MS) with a LOD of 7 μg kg^−1^ for FB_1_, of 3 μg kg^−1^ for FB_2_, and of 3 μg kg^−1^ for FB_3_. Spiked maize, barley, oat, and millet samples with FB_1_ (0 μg kg^−1^, 20 μg kg^−1^, 100 μg kg^−1^, and 500 μg kg^−1^) were tested using the IATC and the commercial ELISA test kit. The results of the IATC and ELISA test kit are summarized in Table 1. The IATC and the commercial ELISA test kit provided consistent results for the spiked cereal samples.

At the same time, the developed IATC assay with a clean-up column had a more sensitive LOD (20 μg kg^−1^) than the commercial ELISA test kit (100 μg kg^−1^) in the cereal samples. The developed IATC assay had a shorter and more convenient test procedure than the commercial ELISA test kit, as shown in Table 2. The IATC, the addition of a high concentration of sodium chloride, and the use of a silica-C18 clean-up column rapidly eliminated the matrix interferences and no large equipment was needed. However, the sample extraction solution required a process of centrifugation and a high ratio of dilution for the commercial fumonisin ELISA test kit. In addition, the IATC assay only needed an incubation of 1 min and a shorter wash process. In contrast, the commercial fumonisin ELISA test kit required a time-consuming incubation process; the incubation and wash needed to take 45 min. For the IATC assay, the color development process only needed to take 4.5 min, and the result could be judged directly with the naked eye without any instrument. However, for the commercial fumonisin ELISA test kit, the color development took 10–15 min and the measure of absorbance values and data analysis needed at least 5 min by instrument. Finally, the total test time required for the IATC (approximately 10 min) was far less than that required when using the commercial fumonisin ELISA test kit (approximately 100 min). Thus, the developed IATC assay combining clean-up column in our study was simpler, quicker, and more sensitive than the commercial ELISA test kit.

### 2.5. Determination of Fumonisins in Real Maize Samples

**Figure 7:**
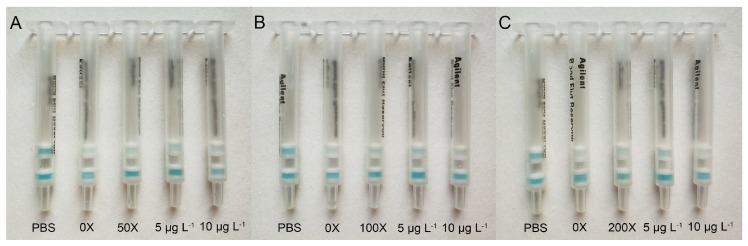
Analysis of the real maize samples with the IATC. (**A**) Maize sample No. 1; (**B**) maize sample No. 2; and (**C**) maize sample No. 3. The detection results from left to right are PBS, sample extracted solution without any dilution, sample extracted solution with corresponding dilution, 5 μg L^−1^ of FB_1_ standard solution, and 10 μg L^−1^ of FB_1_ standard solution. (50×) The maize sample No. 1 extracted solution was diluted 50-fold and analyzed by the IATC; (100×) the maize sample No. 2 extracted solution was diluted 100-fold and analyzed by the IATC; and (200×) the maize sample No. 3 extracted solution was diluted 200-fold and analyzed by the IATC.

Unknown maize samples were analyzed simultaneously using the IATC and LC-MS/MS to evaluate the practicability of the developed assay. The detection results are shown in Figure 7 and Table 3.

A blue color appeared in the test layer and the control layer at the same time when detecting the control solution (PBS) without fumonisins. The real maize sample was extracted with a two-fold extraction ratio (2 g samples were extracted in 4 mL extraction solution). For the undiluted maize sample extracted solution (0×), No blue color was observed on the test layer of the test column for maize samples No. 1, No. 2, and No. 3, demonstrating that fumonisin content in each maize sample was higher than, or equal to, the visual LOD of the IATC for the maize sample (20 µg kg^−1^). As we did not know the concentration of FBs in the real maize sample, each maize sample extracted solution needed to be diluted at running dilutions, then each diluted sample extracted solution needed to be detected subsequently by the IATC, and each test result needed to be compared with that result of the FB_1_ standard solution in order to estimate the content of FBs in the real maize sample. As shown as Figure 7A, a weak blue color was observed on the test layer of the test column at a 50-fold dilution of sample extracted solution in comparison with test column at 10 μg L^−1^ of FB_1_ standard solution (the visual LOD of IATC for FB_1_ in PBS), indicating that the total content of FB_1_, FB_2_, and FB_3_ in maize sample No. 1 was lower than 1000 μg kg^−1^. However, its blue color intensity was weaker than that on the test layer of the test column at 5 μg L^−1^ of the FB_1_ standard solution, which illustrated that the total content of FB_1_, FB_2_, and FB_3_ in maize sample No. 1 was higher than 500 μg kg^−1^. Therefore, the total content of FB_1_, FB_2_, and FB_3_ in maize sample No. 1 detected by the developed IATC assay ranged from 500 to 1000 μg kg^−1^. In the same way, the total content of FB_1_, FB_2_, and FB_3_ in maize sample No. 2 (Figure 7B) ranged from 1000 to 2000 μg kg^−1^, and the total content of FB_1_, FB_2_, and FB_3_ ranged from 2000 to 4000 μg kg^−1^ for maize sample No. 3 (Figure 7C).

As shown in Table 3, the detection results from the developed IATC assay agreed well with the results from the LC-MS/MS analysis, reflecting the good practicability of the developed IATC assay to detect the total content of FB_1_, FB_2_, and FB_3_ in real cereal samples.

## 3. Conclusions

In our study, the developed IATC assay proved to be a simple, rapid, and sensitive way to detect fumonisins in cereal samples without the need for special instruments. The visual LOD of this method was 10 μg L^−1^ for FB_1,_ FB_2_, or FB_3_ in the assay buffer. The developed method could be applied to detect the total concentration of FBs (FB_1_ + FB_2_ + FB_3_), and had a good specificity. The maize sample was extracted and analyzed by the developed IATC directly, and the visual LOD was 20 μg kg^−1^. For the barley, oat, and millet samples, the addition of a high concentration of sodium chloride and the use of a silica-C18 clean-up column could eliminate the sample matrix influences. The visual LOD of the IATC assay in the barley, oat, or millet samples was 20 μg kg^−1^. The IATC assay and the commercial fumonisin ELISA test kit gave consistent results for the analysis of the spiked cereal samples. The total contents of FB_1_, FB_2_, and FB_3_ in real maize samples detected by the developed assay agreed well with the results obtained by LC-MS/MS, reflecting the good practicability of this assay for detecting fumonisins in real samples. The IATC assay proved more sensitive and rapid; in particular, the LOD for the cereal samples (20 μg kg^−1^) showed that the IATC assay was five-fold more sensitive than the commercial ELISA test kit (100 μg kg^−1^) and that the IATC test time (10 min) was approximately one-tenth that of the commercial ELISA test kit (100 min). With a simple sample pretreatment procedure and a rapid analysis process, the developed IATC assay allows for rapid, qualitative, and semi-quantitative detection of the total content of FB_1_, FB_2_, and FB_3_ in cereal samples on-site.

## 4. Materials and Methods

### 4.1. Materials and Buffers

Fumonisin B_1_ (FB_1_), fumonisin B_2_ (FB_2_), fumonisin B_3_ (FB_3_), aflatoxin B_1_ (AFB_1_), ochratoxin A (OTA), zearalenone (ZEN), T-2 toxin standards, keyhole limpet hemocyanin (KLH), and 3,3′,5,5′-tetramethylbenzidine (TMB) were purchased from Sigma-Aldrich (St. Louis, MO, USA). Sodium periodate and glutaraldehyde was purchased from Alfa Aesar (Shanghai, China). CNBr-activated Sepharose 4B gel and horseradish peroxidase (HRP) were purchased from GE Healthcare (Uppsala, Sweden). Anti-HRP antibody was purchased from Beijing Zoman Biotechnology Co., Ltd. (Beijing, China). Dimethyl sulfoxide (DMSO) was purchased from E. Merck (Darmstadt, Germany). Polyethylene frits and 1-mL tubes (SPE cartridges) were purchased from Agilent Technologies (Santa Clara, CA, USA). Silica-C18 (diameter 0.040 mm), NH_2_-derived silica (diameter 0.040 mm), and strong anion exchange silica (SAX) (diameter 0.040 mm) were purchased from Acchrom Technologies (Beijing, China). A fumonisin B_1_ ELISA test kit was purchased from Reagen LLC (Moorestown, NJ, USA). Fumonisin immunoaffinity columns were purchased from Pribolab (Singapore, Singapore).

Phosphate-buffered saline (PBS, pH 7.4, 0.01 mol L^−1^) was used as the assay buffer, phosphate-buffered saline containing 0.05% Tween (PBST, pH 7.4, 0.01 mol L^−1^) was used as the wash solution. Coupling buffer (pH 8.3, NaHCO_3_ buffer containing 0.5 mol L^−1^ of NaCl), blocking buffer (pH 8.0, coupling buffer containing 0.2 mol L^−1^ of glycine), acetate buffer (pH 4.0, containing 0.5 mol L^−1^ of NaCl) was used to prepare the gels, and TMB substrate solution was prepared by adding 4.5 mg of TMB in 450 μL of DMSO to 14.6 mL of phosphate citrate buffer (0.1 mol L^−^^1^ citric acid + 0.2 mol L^−^^1^ Na_2_HPO_4_; pH 4.3) containing 3.25 μL of a 1.29 mol L^−^^1^ H_2_O_2_ solution for chromogenic reaction. Carbonated buffer (pH 9.5, 0.05 mol L^−1^), lysine solution (1 mol L^−1^), sodium periodate solution (1 mol L^−1^), and sodium borohydride solution (1 mol L^−1^) were used to prepare the immunogen FB_1_-KLH and the enzyme conjugate FB_1_-HRP.

### 4.2. Preparation of Antibody

According to the glutaraldehyde method, the immunogen FB_1_-KLH was synthesized as follows: 40 μL of 50% glutaraldehyde was added slowly into the KLH solution (10 mg of KLH was dissolved in 400 μL of 0.01 mol L^−1^ PBS, pH 7.4), and the mixture was stirred at 4 °C for 14 h. The solution was dialyzed against PBS (pH 7.4, 0.01 mol L^−1^) overnight to remove excess glutaraldehyde. Then, 500 μL of FB_1_ solution (1 mg of FB_1_ was dissolved in 500 μL of 0.05 mol L^−1^ carbonated buffer, pH 9.5) was mixed with the above activated KLH solution, and the mixed solution was incubated at 4 °C overnight. Subsequently, 100 μL of lysine (1 mol L^−1^, pH 7.0) was added and the reaction was continued for 3 h. Finally, the resuting immunogen was dialyzed against PBS (pH 7.4, 0.01 mol L^−1^) for 72 h and stored at 4 °C until use.

Two New Zealand white rabbits were each immunized subcutaneously six times with the immunogen FB_1_-KLH at two-week intervals. For the initial immunization, 1 mL of the immunogen (1 mg mL^−1^) mixed with 1 mL of Freund’s complete adjuvant to immunize each rabbit. And 1 mL of Freund’s incomplete adjuvant was used instead of complete to emulsify with an equal volume of the immunogen (1 mg mL^−1^) in the subsequent boost immunizations of each rabbit. The rabbits were bled 10 days after the final immunization to collect the antisera. Finally, the antisera were purified by protein A-Sepharose 4B affinity chromatography to obtained the anti-FB_1_ antibody.

### 4.3. Preparation of Enzyme Conjugate (FB_1_-HRP)

According to the periodate method [29], the enzyme conjugate FB_1_-HRP was synthesized as follows: 2 mg HRP in 500 μL sodium periodate solution (1 mol L^−1^) was activated for 20 min at room temperature; then, 1 mL of FB_1_ solution (1 mg of FB_1_ was dissolved in 1 mL of 0.05 mol L^−1^ carbonated buffer, pH 9.5) was dropwise added into the solution described above, and the mixture was allowed to react for 2 h at room temperature under magnetic stirring. Then, 100 μL sodium borohydride solutions (1 mol L^−1^) were added, and the mixture was incubated at 4 °C for 1 h to terminate the reaction. Finally, the resulting enzyme conjugates were dialyzed against PBS (pH 7.4, 0.01 mol L^−1^) for 72 h. The resulting products were stored at 4 °C until use.

### 4.4. Preparation of Anti-FB_1_ Antibody Gel and Anti-HRP Antibody Gel

The anti-FB_1_ antibody was coupled to CNBr–activated Sepharose 4B gel as described previously [24]. A quantity of 0.5 g gel powder was suspended and swollen in 40 mL HCl (1 mmol L^−1^) for 15 min and then washed using 200 mL HCl (1 mmol L^−1^) in a sintered glass filter. A total of 400 μL of anti-FB_1_ antibody (1.27 mg mL^−1^) was diluted in 2 mL of coupling buffer, and the anti-FB_1_ antibody solution was added to the gel. The mixture of anti-FB_1_ antibody and gel was shaken at room temperature for 2 h. After washing away any residual antibody using a coupling buffer, 10 mL of blocking buffer was added to the coupled anti-FB_1_ antibody gel, which was then shaken for 2 h at room temperature to block the active groups. Finally, the anti-FB_1_ antibody gel was washed three times using acetate buffer and coupling buffer to clean the residual glycine, respectively. The prepared anti-FB_1_ antibody gel was stored at 4 °C after diluting with 5 mL PBS and adding 0.03% Proclin 300.

Anti-HRP antibody gel was prepared as per the preparation of the anti-FB_1_ antibody gel described above, and rabbit anti-HRP antibody (500 μL, 1 mg mL^−1^) was used instead of anti-FB_1_ antibody.

### 4.5. Preparation of Blocked Gel

One gram of gel powder was suspended and swollen in 80 mL HCl (1 mmol L^−1^) for 15 min and washed using 200 mL HCl (1 mmol L^−1^); 10 mL of blocking buffer was then added, and the mixture was shaken for 2 h at room temperature to prepare the blocked gel. Finally, the blocked gel was washed with acetate buffer and coupling buffer three times to remove any residual glycine, respectively. The prepared blocked gel was stored at 4 °C after diluting with 10 mL PBS and adding 0.03% Proclin 300.

### 4.6. Assembly of IATC

The IATC was assembled as described by Yuan [24] including the control layer (on the bottom of SPE cartridge) and the test layer (on the 0.5 cm above the control layer). First, a polyethylene frit was pushed into the bottom of the cartridge, 150 μL mixtures of the anti-HRP antibody gel and blocked gel were added and needless PBS in the mix gel was pushed by a plunger, then the second frit was fixed. Second, the third frit was placed approximately 0.5 cm above the second frit, 150 μL mixtures of the anti-FB_1_ antibody gel and blocked gel were added and needless PBS in the mix gel was pushed by a plunger, and then the fourth frit was fixed. Finally, the assembled IATC was washed twice with PBS to remove any residual gel in the cartridge.

### 4.7. Preparation of Clean-Up Column For IATC

In this study, different solid-phase materials including octadecyl-derived silica (silica-C18), NH_2_-derived silica and strong anion exchange silica (SAX) were used as clean-up sorbents to fill the cartridge. A polyethylene frit was pushed to the bottom of the 1 mL SPE cartridge, 200 mg sorbent was added into the column, and another frit was used to prevent the sorbent floating after adding the sample solution.

### 4.8. LC-MS/MS Analysis

LC-MS/MS analysis was performed according to the Chinese National Standard (GB 5009.240-2016) using a Quattro Premier XE Triple Quadrupole system (Waters, Milford, MA, USA). A Waters ACQUITY UPLC BEH Shield RP C18 LC column (2.1 × 100 mm, 1.7 μm) was used. The mobile phase A was aqueous formic acid (0.1%) and mobile phase B was acetonitrile-methanol solution (50:50, *v*/*v*). The flow rate was 0.35 mL min^−^^1^, the column temperature was 30 °C, and the injection volume was 10 μL. The gradient elution program was: 0 min: 70% A, 2.3 min: 30% A, 4 min: 30% A, 4.2 min: 0% A, 4.8 min: 0% A, 5 min: 70% A. The mass spectrometry detection was carried out in the electrospray ionization positive ion mode (ESI+) and multiple reaction monitoring (MRM) mode with the following parameters: capillary voltage 3.0 kV, source block temperature 120 °C, cone gas 50 L h^−1^, cone voltage 45 V, desolvation temperature 350 °C, desolvation gas 800 L h^−^^1^, collision cell pressure 3.0 × 10^−^^5^ MPa. The quantitative product ions (*m*/*z*) were at 352, 336, and 336 and the collision energies (eV) are at 25, 35, and 35 for FB_1_, FB_2_, and FB_3_, respectively. The qualitative product ions (*m*/*z*) were at 334, 354, and 354 and the collision energies (eV) are at 35, 30, and 30 for FB_1_, FB_2_, and FB_3_, respectively. All fumonisins were quantified by external standard method. Calibration curves and correlation coefficients were *y* = 44.2761*x* + 273.557 (0.9927) for FB_1_, *y* = 37.7222*x* + 176.132 (0.9924) for FB_2_, and *y* = 56.8031*x* + 157.801 (0.9967) for FB_3_, respectively.

### 4.9. Sample Preparation

For LC-MS/MS, 5 g of pulverized cereal samples and 20 mL of acetonitrile-water solution (50:50, *v*/*v*) were added to 50 mL centrifuge tube, and the mixture was shocked for 20 min. The extracted sample was then centrifuged at 4000 rpm for 5 min and the supernatant collected. Two milliliters of supernatant was mixed with 47 mL of PBS with 0.1% Tween-20. The mixture was filtered on microfibre filter and loaded onto the immunoaffinity column with a flow rate of 1–3 mL min^−1^. Then, the column was washed with 10 mL of PBS, and 1 mL of methanol-acetic acid solution (98:2, *v*/*v*) was used to elute the immunoaffinity column three times. The eluent was collected and dried by nitrogen at 55 °C, and the residue was redissolved in 1 mL of acetonitrile-water solution (20:80, *v*/*v*). Finally, the solution was vortexed for 30 s and passed through a 0.22 μm filter and analyzed by LC-MS/MS.

For the IATC, a low concentration of methanol was used to extract FB_1_ from cereal in another study [30], so methanol: PBS (20:80, *v*/*v*) was chosen as the extraction solution in our work. Two grams pulverized cereal samples and 4 mL extraction solution were added to a 25 mL centrifuge tube, the mixture was shocked for 2 min to extract the analytes from the cereal samples. The samples were kept static awhile and the supernatant collected. For the maize sample, the resulting supernatant was analyzed directly by the IATC. For the barley, oat, and millet samples, 2 g of sample with 0.5 g NaCl was extracted, the resulting supernatant was added into a clean-up column with silica-C18 sorbent and the purified solution was pushed out by a plunger. The resulting purified solution was analyzed by the IATC.

### 4.10. IATC Assay Test Procedure

The IATC assay test procedure is shown in Figure 1. This scheme includes two parts: the sample clean-up procedure, and the IATC test procedure. The maize sample had no need of a clean-up procedure, and the barley, oat, and millet sample extracted solutions needed to flow through the clean-up column to remove the matrix interference. Then, 1 mL of sample extracted solution for maize was mixed with the FB_1_-HRP (4 × 10^5^-fold diluted to the sample extracted solution), and 1 mL of the purified sample solution for barley, oat, and millet was mixed with FB_1_-HRP (4 × 10^5^-fold diluted to the purified sample solution). The resultant solution was added into the IATC to immerse the control layer and test layer for 1 min. The IATC was washed three times using PBST (1 mL at a time) and twice using PBS (1 mL at a time), respectively. Finally, the TMB substrate solution (300 μL) was used to immerse both layers for 30 s and pushed out using a plunger. After 4 min, the development of color was observed.

## Figures and Tables

**Table 1:** Analysis of the spiked cereal samples using IATC and commercial ELISA test kit.

Samples	Spiked Concentration (µg kg^−1^)	IATC (*n* = 3)	ELISA Test Kit (µg kg^−1^)
			Means ± SD (*n* = 3)
Maize	0	−, −, − ^1^	ND ^3^
	20	+, +, + ^2^	ND
	100	+, +, +	99.33 ± 2.52
	500	+, +, +	492.31 ± 4.16
Barley	0	−, −, −	ND
	20	+, +, +	ND
	100	+, +, +	99.67 ± 3.06
	500	+, +, +	491.67 ± 4.04
Oat	0	−, −, −	ND
	20	+, +, +	ND
	100	+, +, +	98.34 ± 2.72
	500	+,+, +	494.51 ± 5.86
Millet	0	−, −, −	ND
	20	+, +, +	ND
	100	+, +, +	97.76 ± 1.53
	500	+, +, +	489.62 ± 4.51

^1^ Negative visual result; ^2^ Positive visual result; ^3^ Not detected, the concentration was lower than the limit of detection of the ELISA test kit (100 µg kg^−1^).

**Table 2:** Comparison of the test procedure and LOD of the IATC assay with the commercial ELISA test kit for analysis of cereal samples.

IATC			ELISA Test Kit		
Test Procedure	Test Time	LOD	Test Procedure	Test Time	LOD
Sample extraction	2 min	20 µg kg^−1^	Sample extraction	35 min	100 µg kg^−1^
Clean-up	2 min		Sample adding	5 min	
Incubation and wash	1.5 min		Incubation and wash	45 min	
Color development	4.5 min		Color development	10–15 min	
Measure	- ^1^		Measure	5 min	
Total test time	10 min		Total test time	100–105 min	

^1^ Not applicable.

**Table 3:** Determination of fumonisins in real maize samples by the developed IATC and LC-MS/MS.

Sample	LC-MS/MS (μg kg^−1^)Means ± SD (*n* = 3)				IATC (μg kg^−1^)	
	FB_1_	FB_2_	FB_3_	FBs (FB_1_ + FB_2_ + FB_3_)		FBs (FB_1_ + FB_2_ + FB_3_)
Maize No. 1	574.82 ± 12.70	159.88 ± 6.10	71.23 ± 0.65	805.94 ± 18.95		+ ^1^, 500–1000
Maize No. 2	1057.24 ± 29.33	247.89 ± 18.47	197.51 ± 9.44	1502.65 ± 40.00		+, 1000–2000
Maize No. 3	1917.25 ± 105.50	623.98 ± 13.90	136.64 ± 6.43	2677.88 ± 114.13		+, 2000–4000

^1^ Positive visual result.
